# To the Veterinary Profession in the United States

**Published:** 1884-01

**Authors:** 


### EDITORIAL NOTES.

TO THE VETERINARY PROFESSION IN THE UNITED STATES.—With the new years issue of the Journal of Comparative Medicine and Surgery comes a change in the editorship, Dr. W. H. Porter retiring and Dr. Billings of Boston, whose name is well known to its readers, assuming the duties of associate editor, which have been hitherto so ably fulfilled by the former Dr. Porter withdrew from the Journal on the plea that his many occupations in practice and as lecturer at the Post-graduate school prevented him from devoting to it the time which its interests demanded. With a new hand at the helm comes some new ways and other ideas. It is the purpose of the editors of the Journal to spare neither labor nor expense in endeavoring to make it not only the very best review of its kind in this country, but the best of its kind in the English language. We wish it to be *indeed a Journal* of comparative medicine. *We mean* to *make it a scientifically practically journal.* It must be an exponant of advanced ideas; its work the promotion of professional interests in every possible way. It will be the advocate of no special school, but will try to keep all the schools up to the best standard it can by kind words of criticism and of praise where they can be given. One of the greatest necessities towards making the veterinary profession a power in the land is a better knowledge of the extent of contagious and infectious diseases among our animals. We want statistics, and in this regard desire to open a department in the Journal to be known as "*communications from practice.*" We do not want in this department *reports* of *ordinary cases*, but reports with reference to what comes to pass in each practitioners practice of a contagious, infectious, or suspicious nature; further, any occurances which come to the practitioners observations bearing upon questions of Public Health. What their respective states do in these questions? Whether climatic, telluric or other influences play any part in the extention of such diseases? In other words—How many cases of glanders have you seen this month? What are your state laws and how are they executed with reference to glanders? Do you meet with much tuberculosis among dairy cattle? How many cases? Do you see any evidences that would lead you to think that it is transmissible from animals being stabled together? Evidence of heredity? Remarks with reference to anthrax and anthracoid diseases? Influenza; how many cases? Is it due to a common specific cause; is it a contagious or infectious disease, facts which lead you to form your opinion? *Rabies;* how many cases? Laws with reference to it? Parasitic diseases, such as scab in Sheep? Trichiniasis in pork? Make examinations. These reports will be carfully filed away and a digest made of them and published with each number of the Journal; and as a whole with the first number of the ensuing year. Veterinarians can but see the practical value of the work we desire to do, of which the above is but an intermation. These communications should be sent to Dr. Billings—colleagues having pathological specimens, which they desire to have examined microscopically and reported upon may also send them to Dr. B,—express paid, but when from a distance, they should be carefully put up in some preserving fluid.

Scarlatina Equinæ!—On account of the excitement created in the public mind that the human disease came originally from horses, and on account of the few cases which occur in one man's practise and the very meagre description of the disease in text books, it is necessary to collect all the knowledge possible on the subject, we therefore entreat our subscribers to report every case of this disease as well as the so-called "purpura"—and to pay the greatest attention to the following:

1. Observe carefully the earliest symptoms of disease, whether it appears to occur as a primary complication or in the source of another disease.

2. Observe carefully the conditions of the heart; temperature, and urine. Is albuminuria *always* present or to what extent.

3. Are the dropsical swellings, œdema, due to heart-weakness, or to renal complications?

4. Is it an infectious or contagious disease with all the evidence you can gather.

5. Make inoculative experiments if you can.

7. How does this so-called scarlatina differ from "purpura?"

8. Result of autopsies. Full reports are especially desired.

Trichiniasis in Germany.—Dr. Rupprecht in the *vierteljahrsschrift für gerichtliche medicine* July, 1883, gives some most original ideas as to the cause of trichiniasis in swine that we have yet met with. He considers that the disease was introduced into Germany about 1820, *by English boars, and that these English boars derived it from Chinese hogs* which have been used in the breeding up our present breeds of hogs. He recognizes the fact that trichinæ are *not transmitted;* but fails of any reasonable proof or even argument except that the Chinese people eat rats, and as rats also harbour trichinæ, and and as he is a believer in the rat theory of procine invasion, he thinks, that Chinese hogs and rats, are the cause of trichinosis in English pork and hence in German.

The whole reasoning of this author is of such a visionary character that we should not notice it, except that he attributes the great percentage of trichinosis in American pork to the great number of coolies (Chinaman) employed at the packing houses of Chicago and Cincinnati. "In Amerika, namentlich auch in Chicago und Cincinnati, den mittel punkten der Züchterei und des Exports von Schweinen, *werden viele Hunderttausende Chinesischer Arbeiter (coolies) beschäftigt.*" "*Die Kulis bleiben ihrer Gewohnheit, neben Schweinefleish auch Rattenfleisch zu Verspeisen, auch in Americka true, so dass die Amerikanischen Schweine ungleich mehr Gelegenhei finden, sich mit Trichinen zu inficiren als bei uns.*"

"In America especially in Chicago and Cincinnati, the *the centres of swine-breeding and export of swine, many hundred thousand Chinamen are employed.*"

The "*many hundred thousand Chinamen employed*" by the pork packers of our too large packing centers must be equally surprising to us, and reminds one of the story of the boy who saw "so many cats in the back yard," which was reduced "to our old Tom and another cat."

Just what the Chinese population of this country is I do not know, and the books at my command do not give it, but the whole country has not "*many hundred thousand,*" and it is doubtful if *one single Chinaman,* is employed in the packing houses of Chicago or Cincinnati. Such work is not to their liking.

It is surprising that an editor of Eulenberg's ability should publish an article (in a journal of such a strictly scientific character as the '*Vierteljahrsschift*' in question) which endeavoured to trace the cause of trichiniosis in American pork by such an insane argument as that because "coolies eat pork and rats," American pork must necessarily be more trichinous than any other, *because we employ more coolies.* Really this is the best thing we have read for a long time; the whole argument falls to the ground, because the '*coolies*' are wanting at the packing houses, to supply the connecting link.

Admitting the coolies; the rat theory of infecting hogs has absolutely no foundation. The truth is, where fresh pork scraps are plentiful, as well as the rats to eat them, as in packing houses, the rats are highly trichinous, some eighty per cent. Rats caught in sewers, stables, etc., are scarcely trichinous, say three or four per cent, and scarcely that.

*Again we have no statistics of the proportion of trichiniasis among the celestials at home; not of a single one; neither of Chinese swine or rats*, so that the whole argument falls to the ground as evidently "Grüners-Tisch illusion," which is the same as being visionary in English. B.

